# Genetic diversity of imported PRRSV-2 strains, 2005–2020, Hungary

**DOI:** 10.3389/fvets.2022.986850

**Published:** 2022-10-11

**Authors:** Szilvia Jakab, Eszter Kaszab, Szilvia Marton, Krisztián Bányai, Ádám Bálint, Imre Nemes, István Szabó

**Affiliations:** ^1^Veterinary Medical Research Institute, Budapest, Hungary; ^2^National Laboratory for Infectious Animal Diseases, Antimicrobial Resistance, Veterinary Public Health and Food Chain Safety, Budapest, Hungary; ^3^Department of Pharmacology and Toxicology, University of Veterinary Medicine, Budapest, Hungary; ^4^Veterinary Diagnostic Directorate, National Food Chain Safety Office, Budapest, Hungary; ^5^Hungarian Association for Porcine Health Management, Budapest, Hungary; ^6^National PRRS Eradication Committee, Budapest, Hungary

**Keywords:** *Betaarterivirus suid 2*, molecular epidemiology, phylogenetic analysis, ORF5, vaccine virus

## Abstract

Porcine reproductive and respiratory syndrome virus 2 (PRRSV-2) remains sporadic in Europe. In this study, we investigated the molecular epidemiology of PRRSV-2 infections encompassing 15 years in Hungary. Partial (423 bp long) ORF5 sequences (*n* = 44) from 20 Hungarian pig herds were analyzed. The study strains fell into two genetic lineages, L1 and L5, being L5 strains more prevalent (88.6 vs. 11.4%). Pairwise sequence identities within Hungarian representative PRRSV-2 strains ranged between 84.7 to 100% (nucleotide, nt) and 85 to 100% (amino acid, aa). When compared with reference strains, identity values fell between 87 and 100% (L1, nt 87–91%, aa 87–93%, reference strain IAF-exp91; L5, nt 87–100%, aa 88–100%, reference strain Ingelvac MLV). Epidemiologic examination implied that the majority of L5 strains were imported repeatedly from other European countries where Ingelvac MLV was approved for routine use. The emergence of L1 strains was thought to be associated with a single introduction and subsequent dissemination between pig farms of a large integrator. Results presented here contribute to a better understanding of the epizootiology of PRRSV-2 infections and shed light on the genetic diversity of viral strains in non-endemic countries.

## Introduction

Porcine reproductive and respiratory syndrome (PRRS) has become a major cause of economic losses for the global swine industry since its first description in Europe and North America (1, 2). PRRS virus (PRRSV), the causative agent, is divided into two species within the *Arteriviridae* family, *Betaarterivirus suid 1* and *Betaarterivirus suid 2* (PRRSV-1 and -2, respectively) (3). PRRSVs are enveloped, positive-sense, single-stranded RNA viruses with approximately 15 kb long genome which encodes at least 10 open reading frames (4).

Reproductive failure and respiratory problems in affected sows and piglets are commonly seen irrespectively of the virus species (5), and comparable virulence has been observed in the reproductive disorder among sows (6). However, the respiratory disease is typically more severe in infections caused by PRRSV-2 (7–9).

PRRSV-2 strains have been classified into nine lineages (L1-9) and numerous sub-lineages based on the ORF5 gene (10). This gene is a highly variable region of the genome making it the main target for evaluating phylogenetic relationships among strains. The majority of genetic lineages have been reported to occur in the Americas and Asia (11). On the contrary, in Europe where the enzootic species is PRRSV-1, the diversity of PRRSV-2 is lower and the circulating European PRRSV-2 strains predominantly belong to the sub-lineage 5.1 within L5 (12–18). This sub-lineage is represented by the PRRSV-2 prototype strain VR2332 and its attenuated form, the Ingelvac MLV (also known as RespPRRS) vaccine virus along with their descendants. Beside the prevalent L5 genotype, a few genotype L1 isolates were identified in Slovakia and Hungary, which originated presumably from Canada or the United States (19–22).

In Hungary, the control of PRRS is limited to disease caused by PRRSV-1 and is supported by routine use of different live, attenuated and/or inactivated vaccines, such as Porcilis PRRS, Unistrain PRRS, Reprocyc PRRS EU, Ingelvac PRRS Flex EU, Suvaxyn PRRS and Progressis (23). PRRSV-2 MLV vaccines (including Ingelvac PRRS MLV) have not been registered to use in Hungary.

PRRSV-2 is not widespread in Hungary, and as a consequence, published data about the genetic diversity of Hungarian PRRSV-2 strains is limited (18, 19, 22). This study reports the ORF5 gene based characterization of all Hungarian origin PRRSV-2 strains in a period encompassing 2005–2020.

## Materials and methods

### Samples

From 2005 to 2020, serum and organ samples were collected for routine PRRSV monitoring, or from herds suspected to sustain PRRS disease, as described in detail previously (18, 24, 25). Samples were tested within 48 h of arrival to laboratory by PRRSV-specific PCR or ELISA methods. In case of positive test results, the import licenses and TRACES certificates (provided by the Hungarian veterinary authorities) were inspected to determine the origin of imported pigs or the pig movement between Hungarian farms.

The recorded clinical symptoms associated with PRRSV-2 infection ranged from asymptomatic to mild, but in four outbreaks, severe problems such as abortions, stillborn piglets and respiratory disorders among pre-fatteners with high mortality were documented.

Following PRRSV-specific diagnostic testing the remainders of the serum and organ samples were placed at −20°C and then thawed when molecular epidemiologic investigation was initiated.

### RNA extraction and ORF5 sequencing

RNA was isolated from samples using QIAmp Viral RNA Mini Kit. The amplification of partial ORF5 was performed by nested RT-PCR assay by means of previously published protocols, which are routinely utilized for diagnostic purposes (22, 26). In brief, the first round included reverse transcription followed by PCR using the Qiagen One-Step RT-PCR Kit and the second round was carried out with Top Taq DNA polymerase to amplify a 432 bp long region of the ORF5. The second-round PCR products were sequenced by Sanger method on ABI PRISM 3100 automatic sequencer, or by semiconductor sequencing technology on Ion Torrent PGM (27).

### Sequence analysis

Sequence data were collected from various sources. For the determination of lineages a large ORF5 gene dataset (*n* = 847), which was kindly provided by Dr. Mang Shi, was utilized as reference (10). Furthermore, 86 PRRSV-2 sequences were collected from GenBank deposited by European countries other than Hungary. A total of 37 sequences were newly determined in this study (see Supplementary material, fasta file) and an additional seven GenBank records from Hungary (accession numbers, EF406336, DQ366650, KM514315, MN150539, MN150535, MN150538, MN150540) were added to the analyses. GenBank accession numbers were retrieved for a subset of Hungarian PRRSV-2 strains, which represented unique sequences (accession numbers, ON939096-ON939121).

Multiple alignments of nucleotide and deduced amino acid sequences (corresponding to nt and aa positions 91-522 and 31-174 relative to the ORF5 gene of strain VR2332) were generated by the MUSCLE algorithm implemented in AliView software (28). Pairwise sequence identities were calculated for a subset of sequences in Mega X (29) (such as identities between all Hungarian sequences and the Quebec reference L1 strain, IAF-exp91, the prototype L5 strain, VR2332, and the lineage L5 vaccine virus strain, Ingelvac PRRS MLV (accession numbers, L40898, EF536003, and AF066183). Genetic recombination events were analyzed with program RDP v5.5 (30). Phylogenetic tree of partial ORF5 sequences was built on the IQ-TREE web-server applying the best fit maximum likelihood model (TVM+F+R8) with 1000 ultrafast bootstrap replications (31). Alignment of deduced GP5 amino acid sequences was visually inspected and sequences were compared with relevant reference strains, focusing primarily on the antigenic regions (32–36) and aa sites contributing to virus rebound or escaping from the host immune system (37, 38). Potential N-glycosylation sites (N-X-S/T) in the GP5 were predicted using the NetGlyc 1.0 Server (https://services.healthtech.dtu.dk/service.php?NetNGlyc-1.0).

## Results and discussion

### Classification of Hungarian PRRSV-2 strains

In general, the PRRSV positivity rate between 2005 and 2020 was 10-11% in each year (in total 40,785 positives out of 363,721 tested samples). With the progression of the eradication program this rate reduced gradually (18). Between 2005 and 2020, the presence of PRRSV-2 was confirmed in 11 out of 19 counties involving a total of 20 pig farms (Table 1). PRRSV-2 was detected in nearly all years, although the spatiotemporal distribution of the cases was uneven (Supplementary Figure 1). Following the launch of the National PRRS Eradication Program in 2014, the more intense surveillance activity resulted in an increased detection rate of PRRSV-2.

**Table 1:** PRRSV-2 associated epidemiological and clinical data, Hungary, 2005–2020.

**Pig farms^#^**	**Farm type**	**Sample IDs**	**Year (and lineage)**	**Clinical symptoms**			**Mortality**	**Origin (and year) of animals positive for PRRSV-2^§^**
				**Severity**	**Respiratory**	**Reproduction**		
A	Large-scale farrowing finishing farm	HU12 7703_NEBIH_2013_HU^*^	2005 (L1) 2013 (L1)	Mild Mild to moderate	Yes	Not visible	3% during prefattening period	Pig import from Slovakia (2005, 2013)
B	Large-scale fattening farm	HU21 PRRSV-2_Hungary_102_2012	2006 (L1) 2012 (L1)	Mild	Yes	Not applicable	13% during fattening period	Circulation among herds within Hungary
C	Large-scale fattening farm	4020_NEBIH_2013_HU^*^	2013 (L1)	Mild	Yes	Not applicable	8% during fattening period	Circulation among herds within Hungary
D	Large-scale farrowing prefattening farm	19678_NEBIH_2010_HU^*^ 28663_NEBIH_2011_HU^*^ 27125_NEBIH_2011_HU^*^	2010 (L1) 2011 (L5)	Mild None	Yes	Not visible	2% during prefattening period	Pig import from Slovakia (2011)
E	Large-scale fattening farm	273_NEBIH_2009_HU^*^	2009 (L5)	Mild	Yes	Not applicable	10% during fattening period	Not available
F	Large-scale farrowing finishing farm	23090_NEBIH_2010_HU^*^	2010 (L5)	Mild	Yes	Not visible	10% from weaning to slaughter	Circulation among herds within Hungary
G	Large-scale fattening farm	32708_NEBIH_2011_HU^*^	2011 (L5)	None	Yes	12.7 weaned piglets/farrowing	3.5% during prefattening period	Pig import from Slovakia (2011)
H	Large-scale fattening farm	50708_NEBIH_2014_HU	2014 (L5)	None	Yes	Not applicable	6% during fattening period	Pig import from Slovakia (2014)
I	Breeding farm	11033_NEBIH_2015_HU^*^	2015 (L5)	Stillborn piglets	Only PRRSV infection	90% died newborn piglets rate, at normal farrowing time	No visible effect	Circulation among herds within Hungary
J	Large scale fattening farm	6576_NEBIH_2015_HU^*^ 42487_NEBIH_2016_HU^*^ 60499_56-60_NEBIH_2017_HU^*^ 35565_96-100_NEBIH_2017_HU^*^ 3158_11-15_NEBIH_2017_HU^*^	2015 (L5) 2016 (L5) 2017 (L5)	Mild Mild Mild	Yes	Not applicable	Mortality rate doubles, from 30/month to 75/month	Pig import from Czech Republic (2015), circulation among herds within Hungary (2016, 2017)
K	Large scale fattening farm	59074_NEBIH_2015_HU^*^	2015 (L5)	None	Yes	Not applicable	5% during fattening period	Pig import from Denmark (2015)
L	Large-scale breeding farm	61748_NEBIH_2015_HU^*^	2015 (L5)	None	Yes	Weaned piglets: 11/farrow farrowing rate: 84.78%	11.2% during lactation 2.1% during prefattening and 2% during fattening period	Pig import from Belgium (2015)
M	Large-scale fattening farm	8727_NEBIH_2016_HU	2016 (L5)	None	Yes	Not applicable	6.5% during fattening period	Pig import from Slovakia (2016)
N	Large-scale fattening farm	25004_NEBIH_2016_HU^*^	2016 (L5)	Mortality	Yes	Not applicable	Mortality rate doubles	Pig import from Czech Republic (2016)
O	Large-scale breeding farm	23920_28_NEBIH_2017_HU^*^	2017 (L5)	None	Yes	Not visible	Mortality rate increased among weaned piglets significantly	Circulation among herds within Hungary
P	Fattening farm	32607_NEBIH_2017_HU	2017 (L5)	None	Yes	Not applicable	5.5% during fattening period	Pig import from Denmark (2017)
Q	Large-scale fattening farm	55352_NEBIH_2018_HU 55352_5_NEBIH_2018_HU^*^ 55352_10_NEBIH_2018_HU^*^ 55352_15_NEBIH_2018_HU^*^ 55352_20_NEBIH_2018_HU^*^ 55352_16-20_NEBIH_2018_HU^*^	2018 (L5)	None	Only PRRSV infection	Not applicable	No visible effect	Pig import from Denmark (2018)
R	Large-scale breeding farm	64196_2_NEBIH_2020_HU^*^	2020 (L5)	None	Yes	Farrowing rate 89%, pregnancy rate 91%	Liveborn piglets 13.8/farrow	Circulation among herds within Hungary
S	Fattening farm	47206_125_NEBIH_2020_HU^*^ 47206_150_NEBIH_2020_HU^*^ 46978_4_NEBIH_2020_HU^*^ 46978_1_NEBIH_2020_HU^*^ 46978_11_NEBIH_2020_HU^*^ 40533_NEBIH_2020_HU^*^ 32023_6-10_NEBIH_2020_HU^*^ 107f_NEBIH_2020_HU^*^ 122f_NEBIH_2020_HU^*^ 156f_NEBIH_2020_HU^*^	2020 (L5)	Stillborn piglets	Only PRRSV infection	Liveborn/farrow decreased from 14.28 to 12.53	Mortality during prefattening period increased from 21 to 30 piglets/month	Seed import from Ireland (2020)
T	Fattening farm	56612_81-85_NEBIH_2020_HU^*^ 56612_40-44_NEBIH_2020_HU^*^ 56612_45-49_NEBIH_2020_HU^*^	2020 (L5)	None	Only PRRSV infection	Not applicable	5 died piglets out of 4,000	Pig import from Germany (2020)

# Location of pig farms can be seen in Figure 2.

§ Based on inspection of import licenses and TRACES certificates (provided by the Hungarian veterinary authorities).

* Determined in this study.

Altogether, 44 partial ORF5 gene sequences were obtained from Hungarian pig holdings over a period of 15 years, including the previously characterized isolates (18, 19, 22). The Hungarian sequences shared 84.7–100% nucleotide and 85–100% amino acid identities with each other. By comparing our samples to the IAF-exp91 strain and to the Ingelvac PRRS MLV vaccine virus, 87–91% and 87–100% nucleotide, 87–93% and 88–100% amino acid identities, respectively, were seen. Sequence analyses revealed neither indel mutations, nor recombination events.

**Figure 1:**
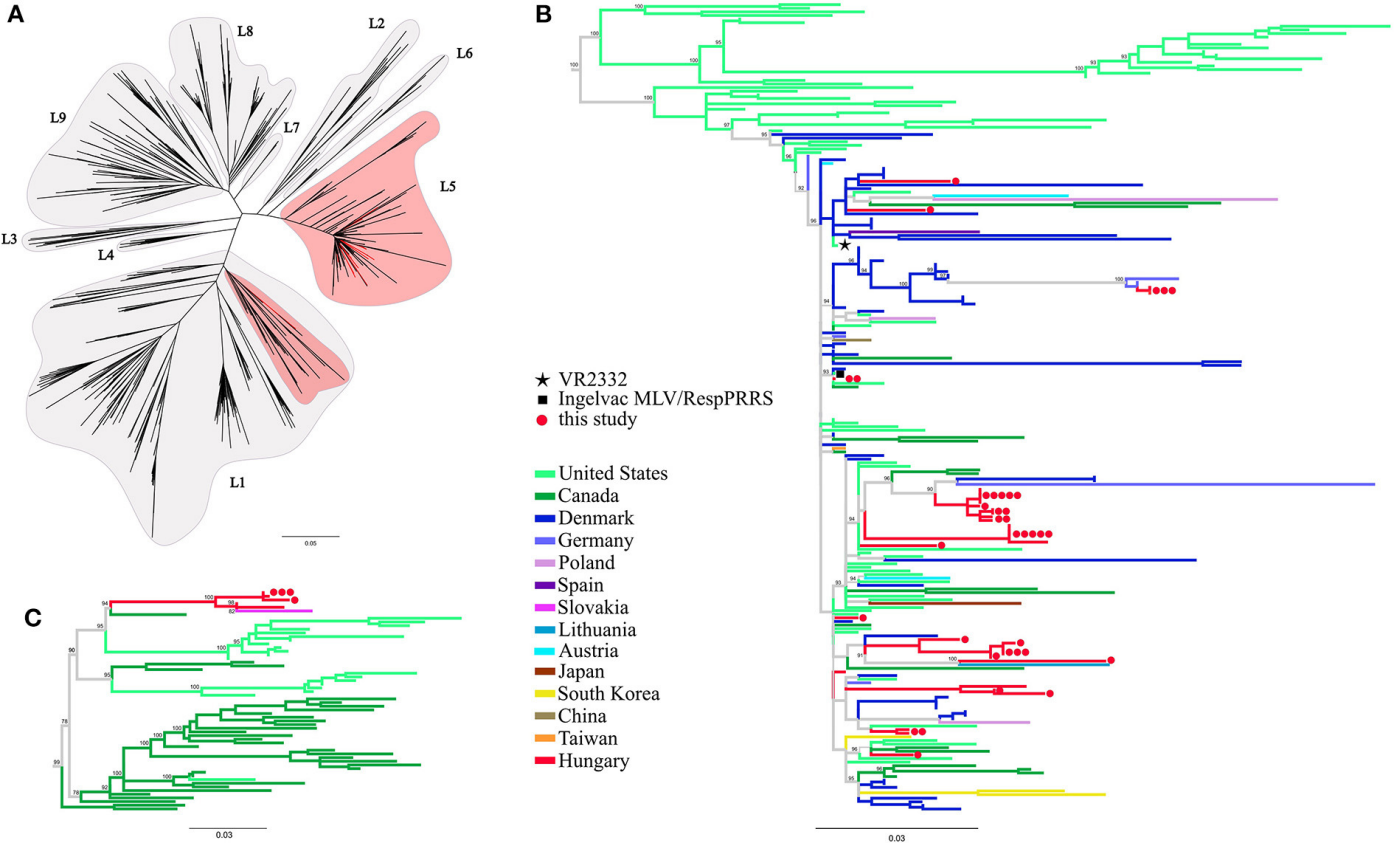
**(A)** Phylogenetic analysis based on global ORF5 sequence dataset including European sequences (*n* = 977) of PRRSV-2. Maximum likelihood method with TVM+F+R8 model and 1,000 ultrafast bootstrap were utilized. **(B)** Lineage 5 and a **(C)** part of lineage 1 sub-trees were expanded for further presentation. The different geographic origin of the sequences were marked with distinct colors. Red circles (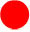) denote the partial ORF5 sequences determined in this study; each dot represents an individual sequence. Prototype strain VR2332 (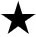) and the vaccine strain Ingelvac MLV (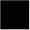) are indicated separately.

Phylogenetic tree reconstruction was performed in order classify the newly determined PRRSV-2 sequences and to verify the relationships of the previously reported records. The nucleotide sequences of this study and the reference dataset of lineages were aligned along with ORF5 sequences from other European countries (Supplementary Table 2). In the dataset 71, 6, 3, 3, 1, 1, and 1 sequences originated from Denmark, Germany, Poland, Austria, Spain, Slovakia and Lithuania, respectively. The strain classification was performed according to Shi et al. (10). Our analyses indicated that all Hungarian PRRSV-2 sequences could be unequivocally classified; i.e. the majority (39/44, 88.6%) typed as L5, while the remainder (5/44, 11.4%) typed as L1 (Figure 1). The ORF5 based phylogenetic tree showed that most of the Hungarian lineage L5 strains are closely related to Danish MLV-related sequences across Europe. The Hungarian L1 variants formed a distinct monophyletic cluster including a Slovakian strain, 36M (39), with no other sequences from additional neighboring countries, which emphasize their unique position among European PRRSV-2 field strains.

### Molecular epidemiology

L5 is a predominant PRRSV-2 lineage worldwide along with L1, L9 and L8. The main geographic locations of lineage L5 strains include Canada, China, USA, Mexico, Korea and Denmark (11). Within lineage L5, it is of great importance to discriminate the Ingelvac PRRS MLV or its descendants from the parental, wild-type VR2332 strain. To date, a particular strain is considered vaccine-related if its ORF5 sequence shares lower than 5% sequence distance from the ORF5 sequence of Ingelvac MLV vaccine virus (10, 11, 40).

**Figure 2:**
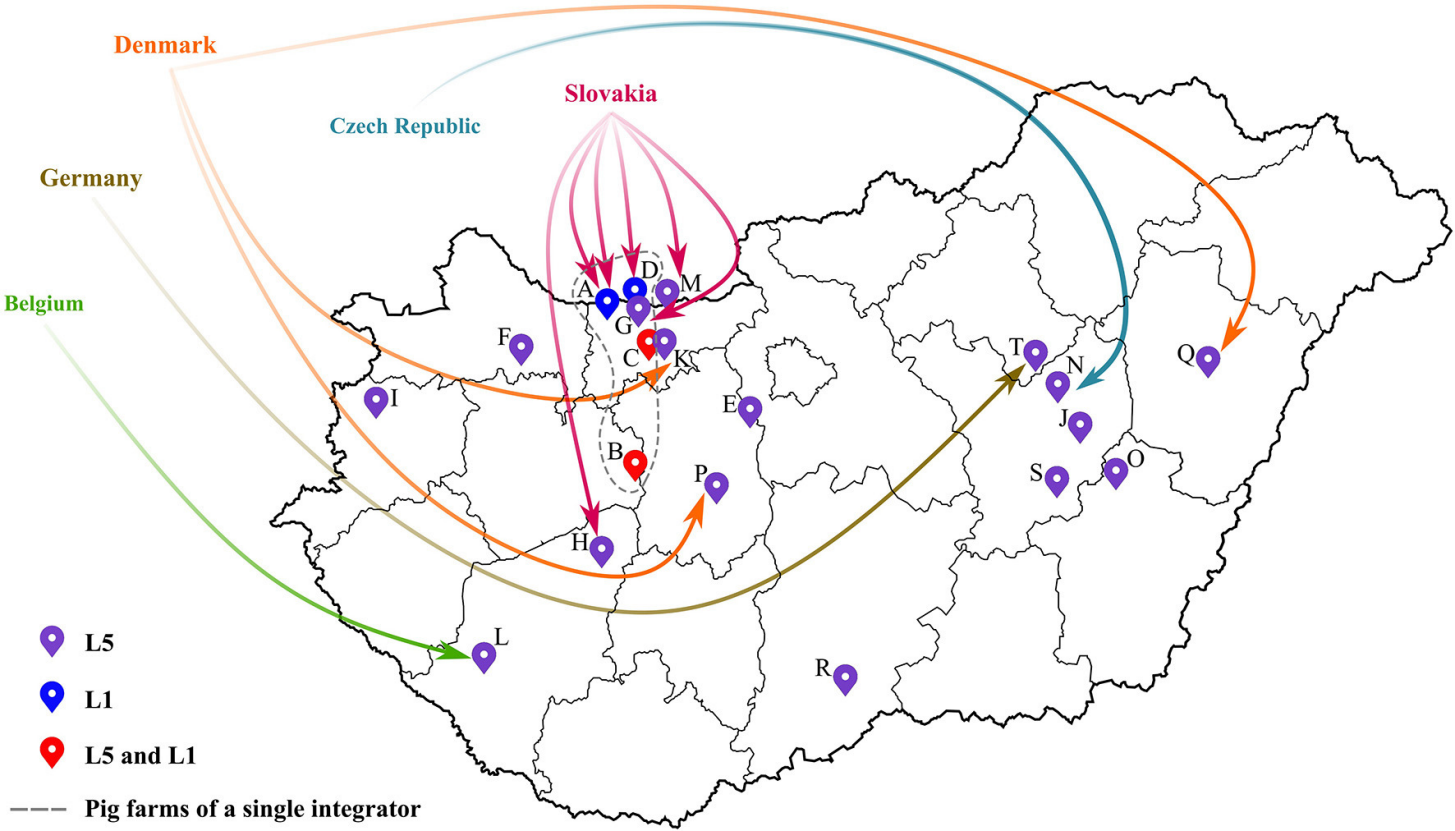
Spatial distribution of all pig farms that were infected with PRRSV-2 from 2005 to 2020. The location of the farms were designated by different colored markers according to the detected lineage of PRRSV-2. Origin of imported fattening stocks were also displayed (for further details the reader is referred to Table 1).

Concerning the Hungarian L5 PRRSV-2 strains (39 samples from 17 pig farms), their partial ORF5 sequences shared 95-100% nucleotide identity with the vaccine virus suggesting that they were vaccine derived viruses. Additionally, as confirmed by observations of field veterinarians, several PRRSV-2 strains were detected after the settling of fattening pigs from abroad—e.g., Belgium, Czech Republic, Germany, Slovakia or Denmark—or could be connected in some way to herd(s) abroad (Figure 2; Table 1). In this regard, it is important to emphasize that Ingelvac PRRS MLV is registered to use in those respective countries, but not in Hungary. PRRSV-2 sequence data are currently scarce in Europe (except Denmark). Yet, sequence and epidemiologic data (together with available import licenses and TRACES certificates) indicated that the imported L5 PRRSV-2 isolates could have originated mainly from Denmark and Slovakia. Although, based on the Hungarian legislation, import solely has been allowed from PRRSV-free herds since 2017, in various cases the animals were reported to arrive from collection stations. In collection stations, pigs from PRRS-infected and -free herds may have been kept in the same air space, a prerequisite that could have favored viral spread between infected and naïve animals. Due to varying regulation of PRRSV-2 vaccination in Europe the Ingelvac MLV vaccine strain could be periodically imported to PRRSV-2 free countries by trading of live animals. Such incidents impose significant risk to Hungarian herds currently declared PRRSV-free, since the PRRS eradication program will come to a completion in 2022. So far, several regulations were made to reduce the risk of reinfection at PRRS free farms of Hungary; the most significant achievement might be that only officially determined PRRS-free stocks are admitted to the country from 2017 onward.

Lineage L1 PRRSV-2 is the most abundant group worldwide; it is mainly dispersed in USA, Canada and China (11). In Hungary, five lineage L1 sequences (from four pig farms) were identified; all five sequences were linked to a single recognized event of introduction dating back to 2005 to a farm of a large integrator and spread between other locations of this integrator between 2006 and 2013 (index farm, A; other farms with subsequent transmission events, B, C, D, and G; Figure 2; Table 1) (19, 22). Epidemiological data suggested that the most likely country of origin for the first recorded L1 strain in Hungary was Slovakia (Figure 2; Table 1). By the end of 2014, the year when the national eradication program in Hungary was launched, PRRSV-2 was eliminated in all farms where L1 strains were previously in circulation (Supplementary Figure 1). Since then no additional L1 strains were identified. In Europe, this particular lineage is represented only by our few characterized sequences and a single Slovakian sample. The phylogenetic analysis further confirmed the hypothesis that these PRRSV-2 strains are wild-type and originated from the USA or Canada (19). The Hungarian and Slovakian lineage L1 PRRSV-2 strains display a unique cluster within Europe and could constitute a country-specific group of L1, a finding also recognized in Korea (41, 42). A more recent extensive study that focused on lineage diversity in USA expanded the representative dataset of L1 sub-lineages (43). According to results of BLAST search, Hungarian L1 sequences shared the greatest sequence identity with a newly defined sub-lineage, L1D-alpha (93%). L1D-alpha contains progenitor isolates and it was predicted to have emerged before 1990 (43, 44). In addition, this sub-lineage presumably predominated the viral population from 1990 to 2004 in the USA (43).

### Analysis of GP5 amino acid sequences

**Figure 3:**
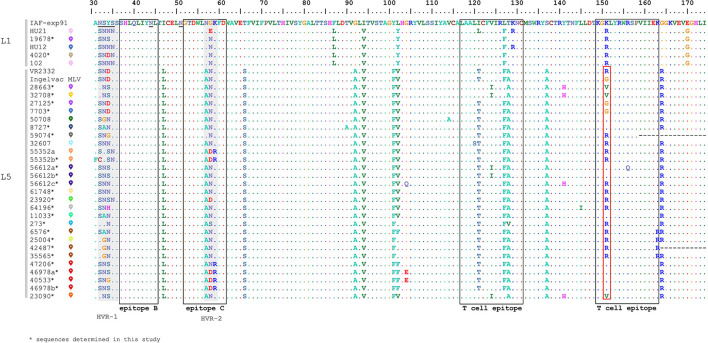
Multiple alignment of the partial GP5 amino acid sequences of all Hungarian isolates of PRRSV-2 from 2005 to 2020 together with reference strain IAF-exp91 and VR2332 (The reader is referred to Supplementary Figure 1 for the colored signs). Epitope sites and highly variables regions are represented by blank boxes and gray boxes, respectively. The site of attenuation of VR2332 is indicated in the red box.

In order to evaluate the diversity of GP5, the non-redundant deduced amino acid sequences were selected from our dataset, resulting in 32 unique variants for subsequent genetic analyses (Figure 3). The Hungarian PRRSV-2 samples showed a relatively high sequence identity within L5 and L1 (proportion of conservative aa sites: 122/144 and 139/144) according to the amino acid alignment of partial GP5 (a protein fragment encompassing aa31-174). Certain aa residues such as aa56, aa57, aa102, and aa104 have already been identified in association with escape mechanisms from homologous neutralization (36, 37). In our case, this finding is particularly relevant for the vaccine related L5 samples. Some of the characterized sequences (such as 25004, 47206, 46978, and 40533) that originated from pig farms with high mortality rate and increased number of stillborn piglets carried mutations compared to Ingelvac MLV, V^102^L/F and G^104^E, which may represent immune escape variants of the vaccine virus. However, these mutations appear in samples of symptom-free pig farms and, for example, not all Danish L5 isolates manifesting clinical signs showed substitutions at these positions (13). Thus, our understanding is limited whether these residues play a role in the evolution of immune escape variants and in the mechanism of virus rebound of the Ingelvac MLV vaccine. Another substantial residue, aa151, is considered to be involved in the attenuation process of VR2332 strain (R → G) (45). We identified numerous substitutions (G^151^K/V/R) in our L5 sequences and half of these reverted to the amino acid of parental strain VR2332 (Figure 3). In fact, high mutation rate and positive selective pressure were found at this position (46, 47).

GP5 is a dominant protein participating in the neutralization process, also it has been acknowledged as a crucial factor contributing to virus rebound (32, 36, 38, 48, 49). Among the recognized antigenic regions, epitope B and C (aa37-45 and aa52-61) were proved to be neutralizing (32–35, 50). Epitope B was completely conserved (^37^SHLQLIYNL^45^) among all Hungarian PRRSV-2 while epitope C, which spans the HVR-2, was variable at position aa57-59. Analyzing the T cell epitopes (aa117-131 and aa149-163) they carried a few substitutions at different amino acid positions.

N-glycosylation of GP5 is a significant mechanism contributing to the infectivity and the antigenicity of PRRSVs and affects the immune response of the host (51–53). Potential N-glycosylation sites were observed at several positions in the GP5 of Hungarian sequences; these include N32, N33, N34, N35, N44 and N51. Two glycosylation sites corresponding to amino acid N44 and N51 seem to be essential for PRRSV infection, therefore these modifications are usually highly conserved in reference (51, 52) as well as in Hungarian PRRSV-2 strains. We found six different N-glycosylation patterns (A-F) composed of three or four asparagines (Supplementary Table 1). The most abundant pattern A (33-44-51) and B (34-44-51) seemed to be widely distributed in the USA (46). N30 is another common glycosylation site (13, 46, 50) which, however, could not be examined from the partial ORF5 sequences of Hungarian PRRSV-2 strains. The N-glycosylation pattern of the full length GP5 regarding Ingelvac MLV vaccine virus strain is 30-33-44-51. Apart from the N30 the Hungarian L5 sequences carry several different changes compared to the vaccine virus, which could imply a trend to evade the host immune response.

## Conclusions

In this study, classification, genetic diversity and distribution of circulating PRRSV-2 strains have been investigated in Hungary, a European country where PRRSV-2 is not enzootic. A dataset containing partial PRRSV-2 ORF5 sequences as well as information about PRRSV-2 positive pig farms were collated and analyzed. Despite the low sample size, the data presented here are thought to give a true overall picture of the epizootiology of PRRSV-2 infections recorded in Hungary between 2005 and 2020, with over 360,000 swine origin samples being tested for PRRSV in this period.

In our study, PRRSV strain surveillance was based on sequencing of the partial ORF5 gene. Although the analysis of partial ORF5 alone may hide some important features of viral evolution, this genomic region is still preferably used in molecular epidemiologic investigations (40, 41, 43). Furthermore, this single-gene based strain characterization approach appeared to be an economically reasonable choice for a pioneering national PRRSV eradication program. Sequencing of the structural region or the whole genome of PRRSV strains had not been a primary aim of Hungarian authorities when the PRRS eradication program was launched, however, sequencing the whole genome in some cases nicely complemented the current methodology (19, 54). Thus, as massively parallel sequencing technologies become part of routine diagnostic laboratory workflows and the amount of data these technologies generate is easier to analyze, it is expected that such new approaches will replace old ones over time. Ideally, these technologies will permit whole genome sequencing and bioinformatics pipelines at reasonable costs to be performed that will be an added value when compared to current technologies and protocols used in routine viral strain characterization.

PRRSV-2 is not a major cause of porcine reproductive and respiratory syndrome in Europe and with few exceptions PRRSV-2 lineages circulating in this continent have been known only from isolated outbreaks. This epidemiologic landscape markedly influences the number of viral sequences reported from different countries; thus, one need to keep in mind that conclusions of this study concerning the country of origin of isolates may have some limitations, even if the route of animal transport and available sequence information mutually increase the plausibility of our findings. Our study indicate that key features about the epizootiology and evolution of PRRSV-2 could be missing in Europe as the virus falls out the focus of intensive surveillance (12, 13). Hence, it seems a realistic scenario that new virus variants that may cause local epizootics in the future may remain overlooked.

## Data availability statement

The datasets presented in this study can be found in online repositories. The names of the repository/repositories and accession number(s) can be found in the article/Supplementary material.

## Author contributions

SJ, EK, SM, KB, ÁB, IN, and IS: collection and analysis of data. SJ, KB, ÁB, IN, and IS: interpretation of results. SJ, KB, ÁB, and IS: writing the draft manuscript. All authors have agreed to the published version of the manuscript.

## Funding

This work was supported by the National Laboratory for Infectious Animal Diseases, Antimicrobial Resistance, Veterinary Public Health and Food Chain Safety, RRF-2.3.1-21-2022-00001. Prepared with the professional support of the Doctoral Student Scholarship Program of the Co-operative Doctoral Program of the Ministry of Innovation and Technology financed from the National Research, Development and Innovation Fund.

## Conflict of interest

The authors declare that the research was conducted in the absence of any commercial or financial relationships that could be construed as a potential conflict of interest.

## Publisher's note

All claims expressed in this article are solely those of the authors and do not necessarily represent those of their affiliated organizations, or those of the publisher, the editors and the reviewers. Any product that may be evaluated in this article, or claim that may be made by its manufacturer, is not guaranteed or endorsed by the publisher.
