# Corrigendum: Genetic diversity of imported PRRSV-2 strains, 2005–2020, Hungary

**DOI:** 10.3389/fvets.2022.1093884

**Published:** 2022-11-28

**Authors:** Szilvia Jakab, Eszter Kaszab, Szilvia Marton, Krisztián Bányai, Ádám Bálint, Imre Nemes, István Szabó

**Affiliations:** ^1^Veterinary Medical Research Institute, Budapest, Hungary; ^2^National Laboratory for Infectious Animal Diseases, Antimicrobial Resistance, Veterinary Public Health and Food Chain Safety, Budapest, Hungary; ^3^Department of Pharmacology and Toxicology, University of Veterinary Medicine, Budapest, Hungary; ^4^Veterinary Diagnostic Directorate, National Food Chain Safety Office, Budapest, Hungary; ^5^Hungarian Association for Porcine Health Management, Budapest, Hungary; ^6^National PRRS Eradication Committee, Budapest, Hungary

**Keywords:** *Betaarterivirus suid 2*, molecular epidemiology, phylogenetic analysis, ORF5, vaccine virus

In the published article, there was an error in affiliation [3]. Instead of “Department of Pharmacology and Toxicology, University of Veterinary Medical Research, Budapest, Hungary”, it should be “Department of Pharmacology and Toxicology, University of Veterinary Medicine, Budapest, Hungary”.

The authors apologize for this error and state that this does not change the scientific conclusions of the article in any way. The original article has been updated.

## Publisher's note

All claims expressed in this article are solely those of the authors and do not necessarily represent those of their affiliated organizations, or those of the publisher, the editors and the reviewers. Any product that may be evaluated in this article, or claim that may be made by its manufacturer, is not guaranteed or endorsed by the publisher.

