# Supplementary material for: Genetic diversity of imported PRRSV-2 strains, 2005–2020, Hungary
Source: Front Vet Sci. 2022 Oct 11;9:986850. doi: 10.3389/fvets.2022.986850 (PMC9595726; doi:10.3389/fvets.2022.986850)

**Supplementary Table 1.** The location and pattern of putative N glycosylation sites in GP5 among Hungarian PRRSV-2 sequences.

| Glycosylation pattern | N-gly sites | |  |  |  |  | % of total | lineages |
| --- | --- | --- | --- | --- | --- | --- | --- | --- |
|  | N32 | N33 | N34 | N35 | N44 | N51 |  |  |
| A |  | **+** |  |  | **+** | **+** | 34.4% (11/32) | L1, L5 |
| B |  |  | **+** |  | **+** | **+** | 25% (8/32) | L5 |
| C |  |  |  | **+** | **+** | **+** | 12.5% (4/32) | L1, L5 |
| D | **+** | **+** |  |  | **+** | **+** | 9.4% (3/32) | L5 |
| E |  | **+** | **+** |  | **+** | **+** | 9.4% (3/32) | L5 |
| F |  |  | **+** | **+** | **+** | **+** | 9.4% (3/32) | L1 |

**Supplementary Table 2.** Summary of European origin PRRSV-2 ORF5 sequenced used in the phylogenetic analysis.

| **Accession No.** | **Strain** | **Country** | **Year** |
| --- | --- | --- | --- |
| AF095500 | 17704A | Denmark | n.a. |
| AF095501 | 17738B | Denmark | n.a. |
| AF095502 | 17835 | Denmark | n.a. |
| AF095503 | 17839 | Denmark | n.a. |
| AF095504 | 17875 | Denmark | n.a. |
| AF095505 | 17876 | Denmark | n.a. |
| AF095506 | 18013 | Denmark | n.a. |
| AF095507 | 18027 | Denmark | n.a. |
| AF095508 | 18031 | Denmark | n.a. |
| AF095509 | 18033 | Denmark | n.a. |
| AF095510 | 18253 | Denmark | n.a. |
| AF095512 | 18680 | Denmark | n.a. |
| AF095513 | 18683 | Denmark | n.a. |
| AF095515 | 19020 | Denmark | n.a. |
| AF095516 | 19259 | Denmark | n.a. |
| AF095517 | 21192 | Denmark | n.a. |
| AF095518 | 23317 | Denmark | n.a. |
| AF095519 | 17739 | Denmark | n.a. |
| AJ223079 | Danish DK3506-12 | Denmark | n.a. |
| AJ223080 | Danish DK5163-17 | Denmark | n.a. |
| AJ223081 | Danish DK5163-23 | Denmark | n.a. |
| AY615794 | LU534 | Austria | n.a. |
| AY615795 | SE521 | Austria | n.a. |
| AY875854 | 2889 | Austria | n.a. |
| JN651742 | Stendal_V1445/99 | Germany | 1999 |
| JN651746 | BH_95/10-08_NA | Germany | 2002 |
| KC506625 | DK-2010-10-1-1 | Denmark | 2010 |
| KC506628 | DK-2010-10-2-2 | Denmark | 2010 |
| KC506629 | DK-2010-10-2-3 | Denmark | 2010 |
| KC506630 | DK-2010-10-3-3 | Denmark | 2010 |
| KC506632 | DK-2010-10-4-2 | Denmark | 2010 |
| KC506633 | DK-2010-10-4-3 | Denmark | 2010 |
| KC506634 | DK-2010-10-5-2 | Denmark | 2010 |
| KC506635 | DK-2010-10-6-3 | Denmark | 2010 |
| KC506637 | DK-2010-30-8-4 | Denmark | 2010 |
| KC506638 | DK-2011-30-1-31 | Denmark | 2011 |
| KC506639 | DK-2011-30-1-34 | Denmark | 2011 |
| KC506640 | DK-2011-30-1-35 | Denmark | 2011 |
| KC506641 | DK-2011-10-2-1 | Denmark | 2011 |
| KC506643 | DK-2011-030311-2 | Denmark | 2011 |
| KC506644 | DK-2011-030311-3 | Denmark | 2011 |
| KC506645 | DK-2011-030311-4 | Denmark | 2011 |
| KC506646 | DK-2011-30-3-13 | Denmark | 2011 |
| KC506647 | DK-2011-30-3-15 | Denmark | 2011 |
| KC506648 | DK-2011-30-3-20 | Denmark | 2011 |
| KC506649 | DK-2011-30-4-3 | Denmark | 2011 |
| KC506650 | DK-2011-30-4-5 | Denmark | 2011 |
| KC506651 | DK-2011-30-4-7 | Denmark | 2011 |
| KC506652 | DK-2011-10-5-1 | Denmark | 2011 |
| KC506653 | DK-2011-30-6-27 | Denmark | 2011 |
| KC506654 | DK-2012-10-1-5 | Denmark | 2011 |
| KC506655 | DK-2012-10-2-8 | Denmark | 2012 |
| KC506656 | DK-2012-10-2-9 | Denmark | 2012 |
| KC506657 | DK-2012-10-3-1 | Denmark | 2012 |
| KC506658 | DK-2012-30-7-16 | Denmark | 2012 |
| KC506659 | DK-2012-30-7-18 | Denmark | 2012 |
| KC506661 | DK-2004-1-3-Lu | Denmark | 2004 |
| KC506662 | DK-2004-1-4-Lu | Denmark | 2004 |
| KC506665 | DK-2003-1-2 | Denmark | 2003 |
| KC506667 | DK-2003-3-3 | Denmark | 2003 |
| KC506668 | DK-2003-4-1 | Denmark | 2003 |
| KC506669 | DK-2003-5-1 | Denmark | 2003 |
| KC506671 | DK-2008-16-2-4 | Denmark | 2008 |
| KC506672 | DK-2008-16-3-3 | Denmark | 2008 |
| KC522648 | 36M | Slovakia | 2007 |
| KC577601 | DK-2004-3-1 | Denmark | 2004 |
| KC714018 | 11_01105 | Poland | 2011 |
| KC714019 | 11_01109 | Poland | 2011 |
| KC714021 | 639590 | Germany | 2011 |
| KC714022 | 653843 | Germany | 2011 |
| KC714023 | 659551 | Germany | n.a. |
| KC714024 | 660501 | Germany | 2011 |
| KC714027 | Jar-72 | Lithuania | 2010 |
| KC714031 | RJ_J_T | Poland | 2012 |
| KC714034 | SP1 | Spain | n.a. |
| KC862575 | DK-2012-01-11-3 | Denmark | 2012 |
| KC862576 | DK-1997-19407B | Denmark | 1997 |
| KC862577 | DK-2011-030311-1 | Denmark | 2011 |
| KC862578 | DK-2004-1-7-Pl | Denmark | 2004 |
| KC862579 | DK-2010-10-1-2 | Denmark | 2010 |
| KC862580 | DK-2010-10-7-1 | Denmark | 2010 |
| KC862581 | DK-2010-10-2-1 | Denmark | 2010 |
| KC862582 | DK-2008-10-1-3 | Denmark | 2008 |
| KC862583 | DK-2010-10-4-1 | Denmark | 2010 |
| KC862584 | DK-2003-2-3 | Denmark | 2003 |
| KC862585 | DK-2004-2-1 | Denmark | 2004 |

**Supplementary Figure 1.** Geographic and temporal distribution of PRRSV-2 in Hungary. (A) Spatial distribution of all pig farms that infected with PRRSV-2 from 2005 to 2020, the location of the farms were designated by different coloured markers. Temporal distribution of PRRSV-2 (B) L5 and (C) L1 positive pig farms.


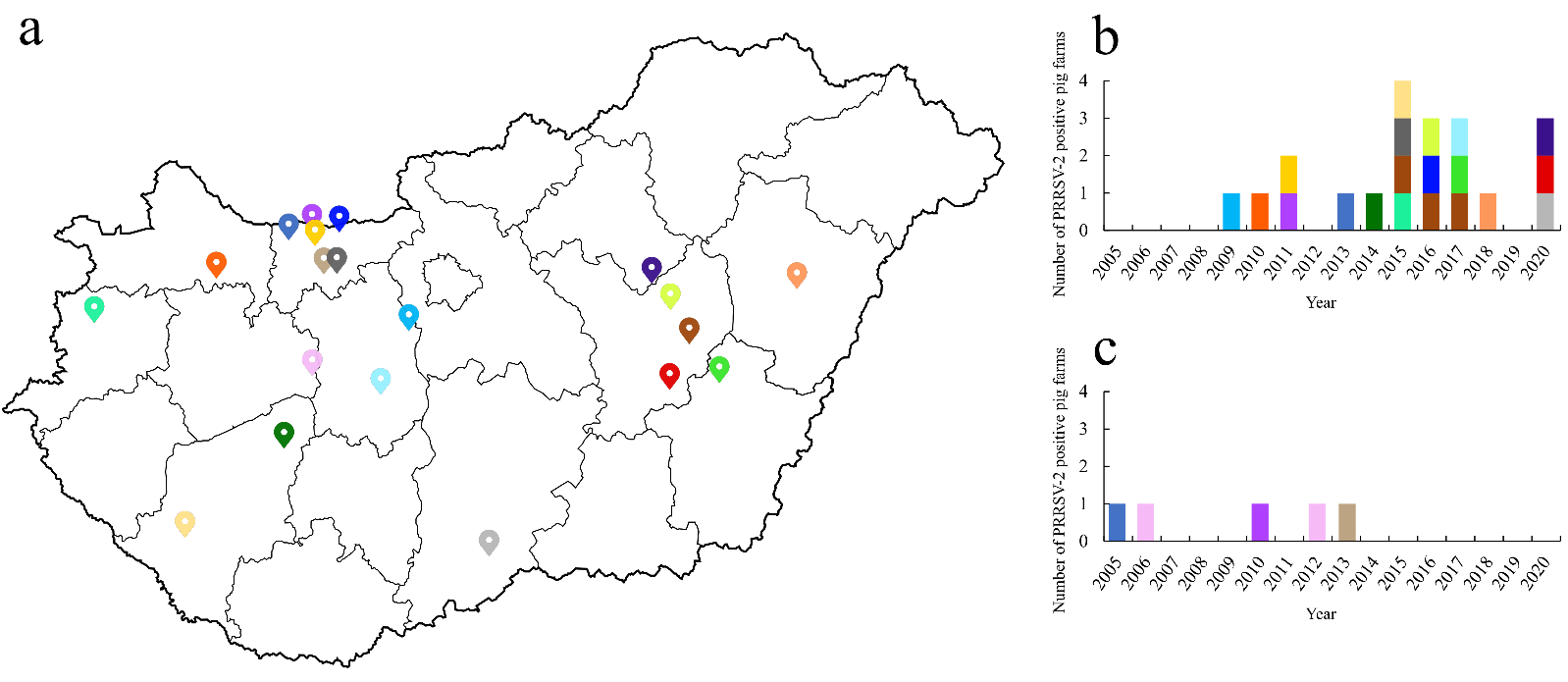

Supplement: Supplementary file 2 [file Data_Sheet_2.docx]
